# Challenges in Using Cultured Primary Rodent Hepatocytes or Cell Lines to Study Hepatic HDL Receptor SR-BI Regulation by Its Cytoplasmic Adaptor PDZK1

**DOI:** 10.1371/journal.pone.0069725

**Published:** 2013-07-23

**Authors:** Kosuke Tsukamoto, Lorenna Buck, Walker Inman, Linda Griffith, Olivier Kocher, Monty Krieger

**Affiliations:** 1 Departments of Biology, Massachusetts Institute of Technology, Cambridge, Massachusetts, United States of America; 2 Biological Engineering, Massachusetts Institute of Technology, Cambridge, Massachusetts, United States of America; 3 Department of Pathology and Center for Vascular Biology Research, Beth Israel Deaconess Medical Center, Harvard Medical School, Boston, Massachusetts, United States of America; University of Navarra School of Medicine and Center for Applied Medical Research (CIMA), Spain

## Abstract

**Background:**

PDZK1 is a four PDZ-domain containing cytoplasmic protein that binds to a variety of membrane proteins via their C-termini and can influence the abundance, localization and/or function of its target proteins. One of these targets in hepatocytes *in vivo* is the HDL receptor SR-BI. Normal hepatic expression of SR-BI protein requires PDZK1 - <5% of normal hepatic SR-BI is seen in the livers of PDZK1 knockout mice. Progress has been made in identifying features of PDZK1 required to control hepatic SR-BI *in vivo* using hepatic expression of wild-type and mutant forms of PDZK1 in wild-type and PDZK1 KO transgenic mice. Such in vivo studies are time consuming and expensive, and cannot readily be used to explore many features of the underlying molecular and cellular mechanisms.

**Methodology/Principal Findings:**

Here we have explored the potential to use either primary rodent hepatocytes in culture using 2D collagen gels with newly developed optimized conditions or PDZK1/SR-BI co-transfected cultured cell lines (COS, HEK293) for such studies. SR-BI and PDZK1 protein and mRNA expression levels fell rapidly in primary hepatocyte cultures, indicating this system does not adequately mimic hepatocytes in vivo for analysis of the PDZK1 dependence of SR-BI. Although PDZK1 did alter SR-BI protein expression in the cell lines, its influence was independent of SR-BI’s C-terminus, and thus is not likely to occur via the same mechanism as that which occurs in hepatocytes *in vivo*.

**Conclusions/Significance:**

Caution must be exercised in using primary hepatocytes or cultured cell lines when studying the mechanism underlying the regulation of hepatic SR-BI by PDZK1. It may be possible to use SR-BI and PDZK1 expression as sensitive markers for the in vivo-like state of hepatocytes to further improve primary hepatocyte cell culture conditions.

## Introduction

The risk of atherosclerosis, a major cause of coronary heart disease (CHD) is inversely proportional to high density lipoprotein (HDL) cholesterol [1], [2]. HDL metabolism is controlled, in part, by its receptor ‘Scavenger Receptor, class B type I’ or SR-BI [3]–[5]. SR-BI (509 amino acids), a member of the CD36 superfamily [6], is an integral membrane, cell surface glycoprotein with two transmembrane domains, relatively short N- (∼7 residues) and C-terminal (∼45 aa) cytoplasmic domains and a large extracellular loop. SR-BI binds to HDL and other lipoproteins [7]–[10] and mediates very efficient cellular uptake of HDL’s cholesteryl esters (CEs) via *a* process called selective lipid uptake’ [7], [11]–[13].It also mediates cellular efflux of unesterified cholesterol [14]. *In vivo*, the greatest SR-BI-mediated selective uptake occurs in the liver and steroidogenic organs where SR-BI is most highly expressed [7], [15]. A minor, alternatively spliced, form of SR-BI, called SR-BII [16] differs from SR-BI only in its C-terminal cytoplasmic domain, whose sequence does not resemble that in SR-BI.

In mice SR-BI, especially hepatic SR-BI, plays a role in many physiologic and pathophysiologic systems [3], [17]–[27], including lipoprotein metabolism, atherosclerosis and coronary heat disease [4], [5], [17], [28]. In endothelial cells SR-BI mediates HDL-dependent signal transduction (e.g., activation of eNOS) via multistep signaling pathways [29], [30]. Murine and human SR-BIs have similar activities and distributions and human SR-BI influences human HDL metabolism [3], [31], [32]. In addition, human hepatic SR-BI is a co-receptor for hepatitis C virus [25]–[27] and possibly malaria [33], [34].

In the liver, the four PDZ-domain containing protein PDZK1 binds to SR-BI’s C-terminus (mouse sequence: EAKL) and is responsible for the post-transcriptional control of SR-BI’s location and stability [35]–[37]. In PDZK1 KO mice, there is an ∼95% reduction in hepatic SR-BI protein expression, but no reduction in steroidogenic tissues, macrophages, or lung-derived endothelial cells [36], [38]. Thus, PDZK1 is a tissue-specific regulator of SR-BI and hepatic SR-BI has a distinctive requirement for PDZK1. As a consequence, alterations in hepatic PDZK1 expression can dramatically influence plasma HDL metabolism and structure, as well as atherosclerosis and coronary artery disease 36,38–40. Furthermore, in endothelial cells, PDZK1 binding to SR-BI mediates an HDL/SR-BI/PDZK1/eNOS signaling pathway [29], [30], [40]. PDZK1 also binds to the carboxy termini of other membrane proteins, including ion channels and transporters (e.g., CFTR) and cell surface receptors [40], [41]. PDZK1 does not appear to bind to the C-terminus of SR-BII (mouse sequence: SAMA) [16], [36].

The mechanism by which PDZK1 controls the levels and surface expression of hepatic SR-BI is not well understood. Some of the features of PDZK1 required to control hepatic SR-BI have been identified in studies involving hepatic expression of wild-type and mutant forms of PDZK1 in wild-type (WT) and PDZK1 KO transgenic mice [37], [40], [42]–[45]. Because the generation and analysis of PDZK1 transgenic animals is time consuming and expensive and does not readily permit analysis of the detailed molecular and cellular mechanisms underlying the regulation of hepatic SR-BI by this adaptor, a simple cell culture system for probing these mechanisms is highly desirable. Ideally, an appropriate hepatocyte cell culture system, such as primary hepatocytes in culture, that exhibits the PDZK1-dependent SR-BI activity seen *in vivo* would be an ideal system for such studies. One major concern with *in vitro* primary hepatocyte cultures is their propensity to lose their differentiated phenotype upon dissociation from the liver [46]–[48]. Certain culture conditions, such as sandwich culture configurations and 3D perfused cultures, have resulted in improved maintenance of cell morphology, protein synthesis, and metabolic capacity [47], [49], [50], but these approaches have largely been demonstrated for rat and human cells, and relatively little appears in the literature regarding conditions that enable long term culture of highly functional mouse hepatocyes, especially those from C57Bl mice (Buck et al, unpublished data). Several investigators have previously reported that expression of PDZK1 as a transgene in cultured cells can alter the levels of SR-BI in those cells [26], [35], [51]. Here we examined the potential of studying the mechanisms underlying PDZK1’s regulation of hepatic SR-BI using two cell culture systems. The first used primary mouse hepatocytes in a ‘sandwich’ culture protocol designed to retain in vivo activities by precisely controlling pericellular oxygen concentration (Buck et al, unpublished data). Mouse hepatocytes cultured in this fashion maintain both higher sensitivity to killing by acetaminophen and rates of albumin secretion that are greater than those previously reported for other methods of culturing primary mouse hepatocytes (Buck et al, unpublished data). The second system used either COS or HEK293 cell lines co-transfected with cDNA expression vectors for SR-BI and PDZK1. Unexpected problems arose using both types of systems, suggesting that caution must be exercised in using these systems to study the regulation of hepatic SR-BI by PDZK1.

## Results and Discussion

### Regulation of SR-BI Expression in Cultured Primary Hepatocytes

**Figure 1:**
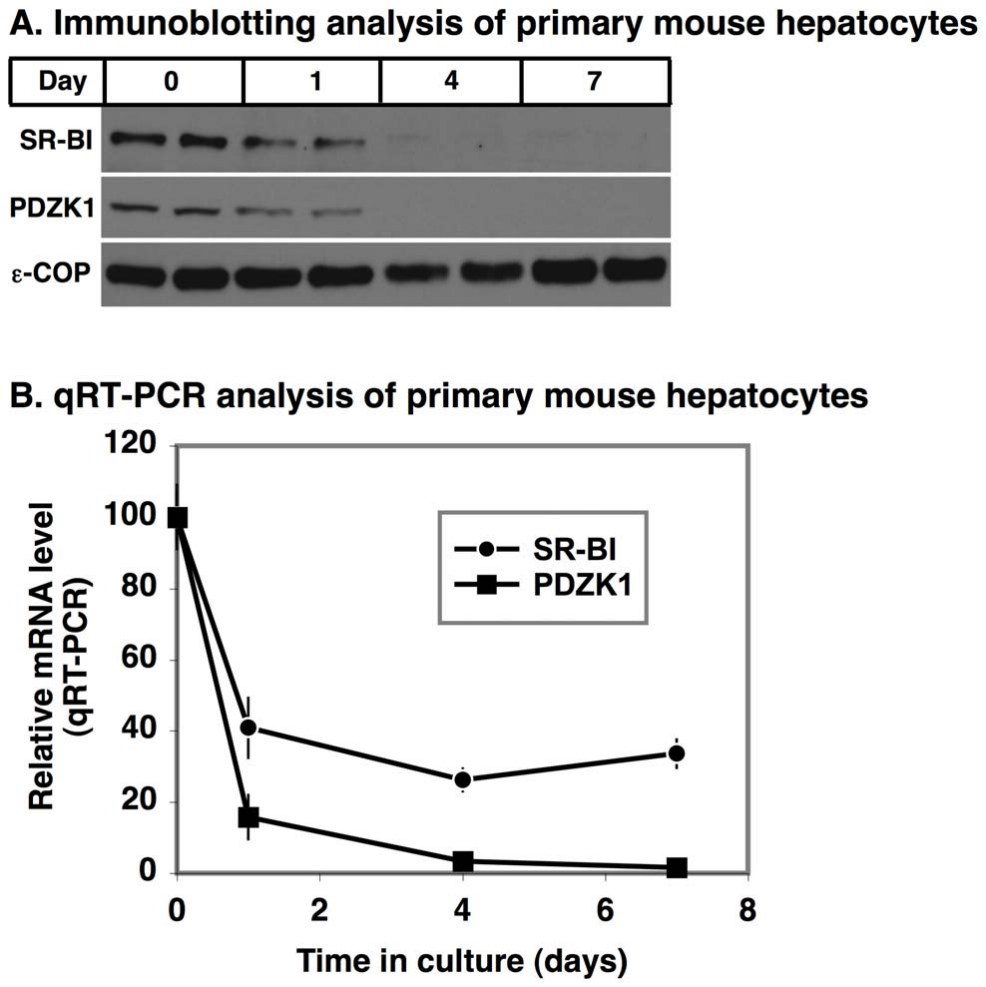
Time course of expression of SR-BI and PDZK1 in cultured primary mouse hepatocytes. Hepatocytes were isolated from the livers of wild-type mice as previously described in Materials and Methods and [58]. Immediately after isolation some of the cells were lysed (time  = 0) and others were plated at 80,000 cells/cm^2^ onto collagen gels with 3% Matrigel and a 1.3 mm medium depth, as described in Materials and Methods. These cells were subsequently maintained in culture for the indicated times, lysed and lysates were analyzed for protein expression using immunoblotting (A) and mRNA expression using qRT-PCR (B). **A** For protein analysis lysates (20 µg protein) were subjected to SDS-PAGE and immunoblotting with polyclonal anti-SR-BI (mSR-BI^495^), polyclonal anti-PDZK1 and polyclonal anti-ε-COP (loading control) antibodies, and the proteins subsequently visualized using enhanced chemiluminescence detection. **B** For mRNA analysis Trizol lysates were subjected to qRT-PCR analysis and the approximate number of mRNA copies/cell was calculated by normalization to 18S rRNA abundance and expressed as percent of the number of copies at time 0. The average 100% of control copy numbers/cell at time 0 were: SR-BI, 19.3; PDZK1, 6.4.

In an attempt to establish a cell culture system that recapitulates the *in vivo* dependence of hepatic SR-BI on PDZK1, we isolated primary hepatocytes from mice and cultured the cells using a ‘sandwich’ culture method (cells plated on collagen type I gels and supplemented with a thin Matrigel overlay (BD Biosciences) with cell seeding densities and medium volume adjusted to provide a physiologically-relevant oxygen tension (i.e., an oxygen tension comparable to the central zone in the liver sinusoid) at the cell surface). These conditions result in the long-term retention of function as assessed by albumin secretion and sensitivity to killing by acetaminophen (see Methods). Figure 1A shows immunoblotting analysis of SR-BI and PDZK1 protein expression in mouse primary hepatocytes cultured using these newly developed conditions for 2D sandwich culture (Buck et al, unpublished data). Expression of the COPI coat protein ε-COP was also determined as a sample loading control. At the time of hepatocyte isolation (day 0), we detected substantial levels of both SR-BI and PDZK1 proteins. On day 1 of culture, there was a reproducible decrease in their expression levels compared to the ε-COP control, which remained relatively unchanged. By day 4 and 7, SR-BI and PDZK1 protein expression levels were very low or nearly undetectable. [Similar results were observed in preliminary studies of primary rat hepatocytes (not shown).] Figure 1B shows the qRT-PCR analysis of the associated levels of SR-BI and PDZK1 mRNA expression in these cells as a function of time in culture. As was the case for protein expression, the mRNA levels dropped quickly after the cells were placed in culture, although the residual levels of SR-BI mRNA after several days in culture were higher than those for PDZK1.

The mechanism(s) underlying the relatively rapid loss of SR-BI and PDZK1 mRNA and protein expression when primary murine (mouse) hepatocytes are placed in culture is unclear. It is possible that in intact liver the polarity of hepatocytes and/or their distinctive cell-cell and cell-matrix interactions significantly impact the stability of the SR-BI and PDZK1 proteins and/or the expression of their genes. Further, heterotypic cell interactions including both matrix and temporally-regulated secretion of growth factors and cytokines may contribute to hepatocellular function [52], along with systemically-regulated factors that have not yet been clearly identified in the mouse system. The problem of maintaining fully normal *in vivo* phenotypes of hepatocytes in culture has been recognized for years. Substantial progress has been made in developing primary hepatocyte culture conditions to more completely recapitulate their *in vivo* physiology, although different conditions are often optimal for different species and relatively few novel methods have been applied to culture of C57bl-derived liver cells [47], [53]. The conditions used here were chosen as optimal for mouse culture from among those reported in the literature (Buck et al, unpublished data), Nevertheless, SR-BI and PDZK1 protein and mRNA expression levels fell rapidly after the hepatocytes were placed into culture. Thus, our results suggest that the expression of SR-BI and PDZK1 proteins in hepatocytes are particularly sensitive to non-native environments. It may be possible to use SR-BI and PDZK1 expression as surrogate markers for normal hepatocytes to further improve primary hepatocyte cell culture conditions. At this time, however, it appears that our current 2D methods are not adequate to permit robust *in vitro* analysis of the mechanism by which PDZK1 normally influences the cellular localization and expression levels of SR-BI in hepatocytes *in vivo*.

### Regulation of SR-BI Expression in Transfected COS Cells

Because of the limitations of using cultured primary murine hepatocytes for studying the PDZK1-mediated regulation of SR-BI (described above), we turned to stable cells lines (COS and HEK293) in an attempt to study this regulation. For some of these experiments, we plated COS cells on day 0 into 6-well plates (1.5 x 10^5^ cells/well) and on day 1 transfected the cells with 4 µg total DNA per well using cDNA expression vectors encoding SR-BI, PDZK1 or an empty vector control (see Methods). On day 3 we measured protein expression levels (immunoblotting) and receptor activity. In preliminary experiments we transfected COS cells with a 50∶50 mixture (w/w) of the SR-BI expression vector and either the PDZK1 or ‘empty’ control vector. We observed high levels of SR-BI and PDZK1 expression over the very low endogenous background levels, but no effect of the coexpression of PDZK1 on the levels of SR-BI protein expression.

**Figure 2:**
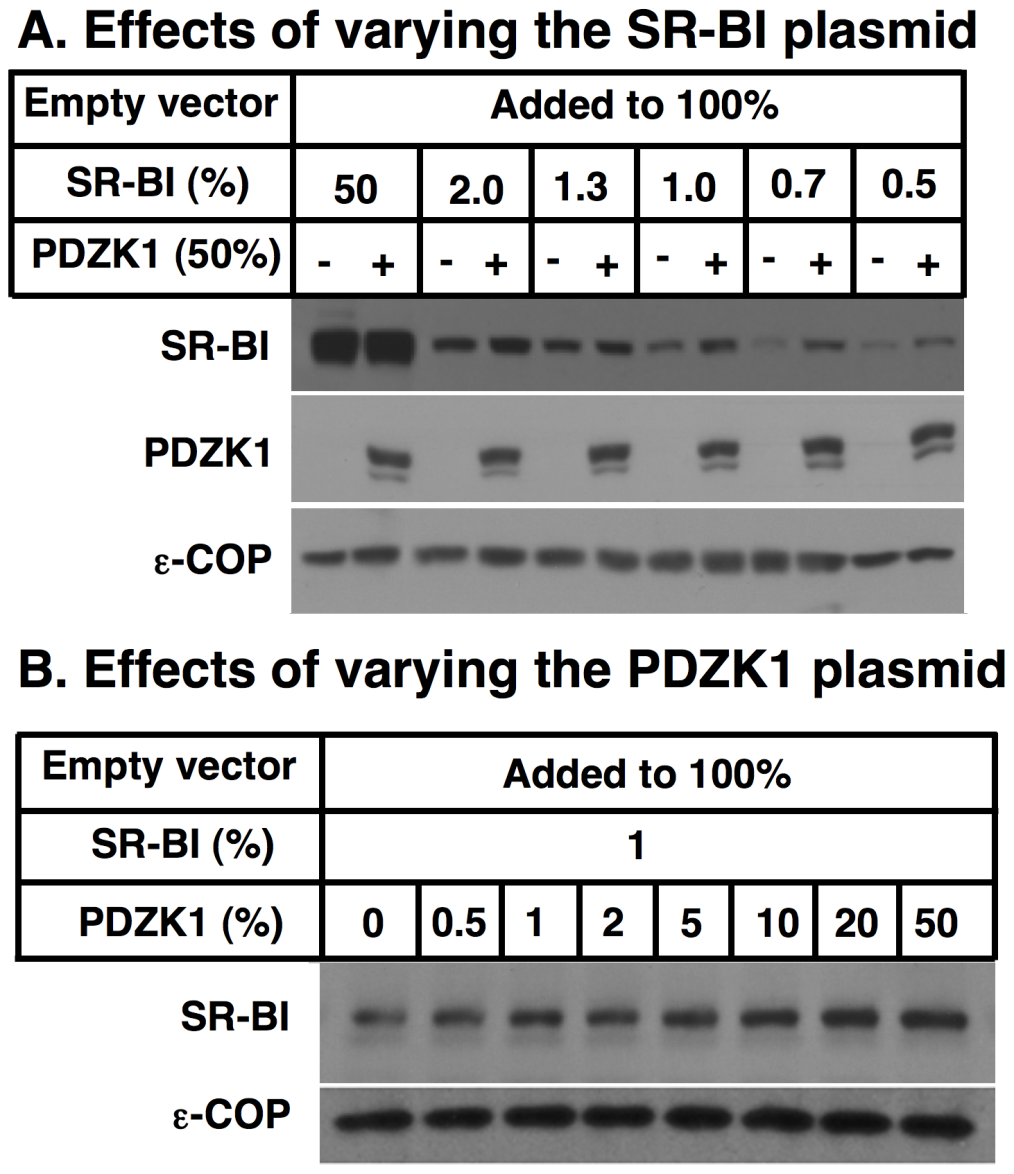
Effects of PDZK1 co-transfection on SR-BI protein levels in COS cells. COS cells were plated into the wells of 6 well plates on day 0 and transiently transfected with a total of 4 µg DNA (100%)/well on day 1 using the indicated plasmids encoding SR-BI, PDZK1 and an empty vector at the indicated relative concentrations (%). On day 3 the cells were harvested, lysed, and lysates (20 µg protein) were subjected to SDS-PAGE and immunoblotting with polyclonal anti-SR-BI (mSR-BI^495^), polyclonal anti-mouse PDZK1 and polyclonal anti-ε-COP (loading control) antibodies. **A** Effects of varying amounts of SR-BI expressing plasmid in the transfection together with either 0% (−) or 50% (+) PDZK1 expressing plasmid. **B** Effects of varying amounts of PDZK1 expressing plasmid transfected together with 1% SR-BI expressing plasmid.

It seemed possible that the high levels of SR-BI expression in this preliminary experiment may have masked any subtle effects of PDZK1 on SR-BI expression. Indeed, we have reported previously that high level hepatic overexpression of an SR-BI transgene in PDZK1 KO mice can restore essentially wild-type levels of hepatic SR-BI protein expression and activity *in vivo* in the absence of hepatic PDZK1 [54]. However, others have reported an influence of PDZK1 on SR-BI expression in transfected cultured cells (26,35,51]. Therefore, we repeated the coexpression experiments using varying amounts of the SR-BI expression vector (50%–0.5% of total transfected DNA) and a fixed amount of the PDZK1 vector (50%) together with sufficient additional empty vector DNA to bring the total transfected DNA per well to 4 µg (100%). Figure 2A shows that substantial expression of transgene encoded PDZK1 protein (middle panel) had essentially no effect on the amount SR-BI protein when the ratio of SR-BI-to-PDZK1 was 50∶50 (two left lanes). However, there was a highly reproducible increase in the amount of SR-BI in the presence of PDZK1 compared to the control when lower levels of SR-BI vector were used (2–0.5% of total DNA, Figure 2A right lanes). A similar result was obtained when HEK293 cells were used in place of COS cells (Fig. S1).

Figure 2b shows the effects on SR-BI protein expression of increasing the relative amounts of PDZK1 expression vector in the transfection (0–50% of total) when the amounts of SR-BI vector were held constant at 1% of the total DNA. There were increased amounts of SR-BI protein seen as PDZK1 vector increased from 0–10% (PDZK1:SR-BI vector ratios of 0–5) with modest additional increase in SR-BI with additional PDZK1 (20 and 50%).

**Figure 3:**
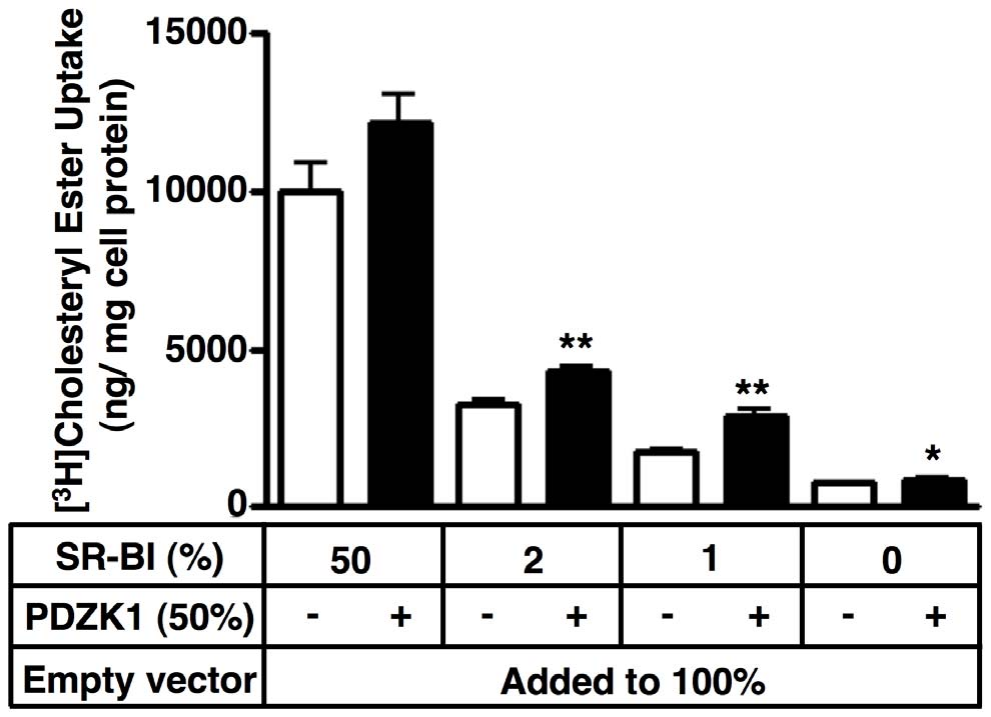
Effects of PDZK1 co-transfection on SR-BI-mediated [^3^H]cholesteryl ester uptake from HDL in COS cells. COS cells were plated into the wells of 6 well plates on day 0 and transiently transfected with a total of 4 µg DNA (100%)/well on day 1 using the indicated plasmids encoding SR-BI, PDZK1 and an empty vector at the indicated relative concentrations (%). On day 1, the cells were harvested, counted and plated into the wells of 24 well plates. On day 2, [^3^H]cholesteryl ester ([^3^H]CE) uptake from [^3^H]CE-HDL (10 mg of protein/mL, 2 hr, 37°C) was determined as described in Materials and Methods. All values represent receptor-specific activities calculated as the differences between activity in the absence (quadruplicate determinations) and presence (duplicate determinations) of a 40-fold excess of unlabeled HDL. Statistical analyses of data obtained with or without co transfection of the PDZK1 plasmid (50%) were performed using the unpaired two-tailed *t* test at 95% confidence intervals (*: p<0.05, **: p<0.005).

To determine if the PDZK1-dependent increase in SR-BI protein expression were functionally relevant, we measured the ability of the transfected COS cells to take up [^3^H]cholesteryl ester ([^3^H]CE) from [^3^H]CE-labeled HDL, a major activity of SR-BI [7]. Figure 3 shows, as expected, that cellular uptake of [^3^H]CE from [^3^H]CE-HDL decreased with decreasing amounts of transfected SR-BI expression vector (and thus expressed SR-BI protein), both in the absence (white bars) and presence (black bars) of cotransfected PDZK1 expression vector. Importantly, the increase in SR-BI cellular protein by cotransfection with PDZK1 at low levels of SR-BI expression (<2% of total transfected DNA, e.g., see Figure 2) was accompanied by increased SR-BI-mediated [^3^H]CE uptake from HDL (compare black and white bars). Thus, the PDZK1-mediated increase in SR-BI protein at low levels of SR-BI expression resulted in increased SR-BI activity, as has been observed in hepatocytes *in vivo* [36], [37], [42]–[44].

The PDZK1-dependence of SR-BI abundance and activity in the transfected COS cells raised the possibility that this *in vitro* cultured cell system might mechanistically mimic the PDZK1-dependence of SR-BI in livers *in vivo*. To explore this possibility, we examined the effects of coexpression in COS cells of PDZK1 on the abundance of three SR-BI variants that do not have PDZK1 binding sites at their C-termini and thus either do not *in vivo* or are not expected to exhibit PDZK1 dependence [36], [45]. Two of these variants are C-terminal deletion mutants of SR-BI lacking either a single amino acid at position 509 (Δ509) or essentially the entire C-terminal cytosolic domain (residues 468 to 509, ‘ΔC-term’). Studies of *the in vivo* hepatic overexpression of a Δ509 transgene indicated that the truncated protein exhibits very low expression and activity, presumably because of its inability to interact with PDZK1 [45]. We also examined a natural splice variant of SR-BI, called SR-BII, whose C-terminal cytoplastic domain is encoded in an alternatively spliced exon and differs completely from that of SR-BI [16]. *In vivo* the expression of hepatic SR-BII is not dependent on PDZK1 and its C-terminal sequence is not expected to bind to the PDZ domains of PDZK1 [36].

**Figure 4:**
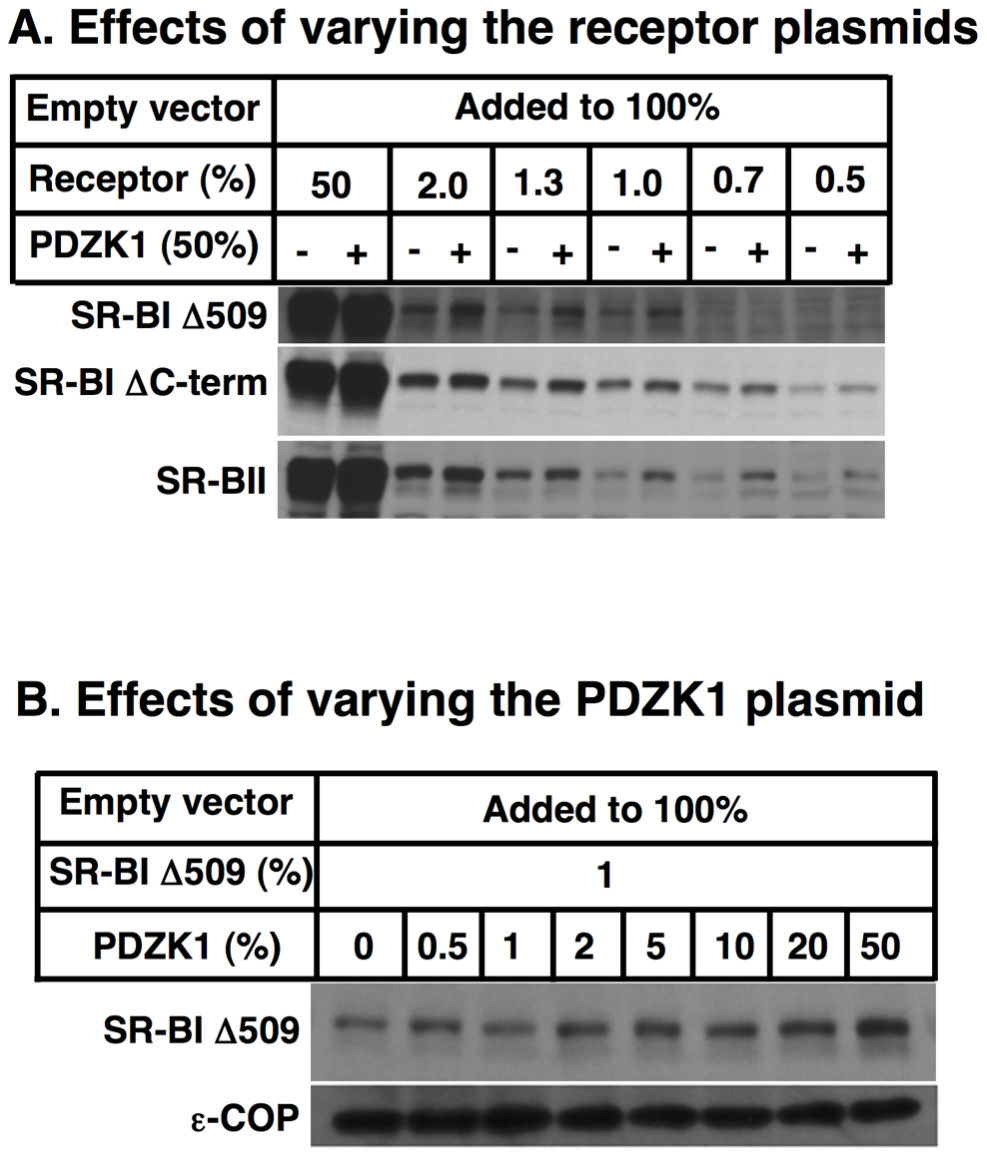
Effects of PDZK1 co-transfection on mutant SR-BI and SR-BII protein levels in COS cells. COS cells were plated into the wells of 6 well plates on day 0 and transiently transfected with a total of 4 µg DNA (100%)/well on day 1 using at the indicated relative concentrations (%) of the indicated plasmids encoding PDZK1, a control empty vector and vectors encoding variants of SR-BI. These variants include SR-BI Δ509 (a mutant lacking a single amino acid at the C- terminus), SR-BI ΔC-term (a mutant lacking essentially the entire C- terminal cytoplasmic domain of SR-BI), and SR-BII (a splicing variant whose entire C-terminal cytoplasmic domain differs from that of SR-BI). On day 3 the cells were harvested, lysed, and lysates (20 µg protein) were subjected to SDS-PAGE and immunoblotting with polyclonal anti-SR-BI (KKB-1) and polyclonal anti-ε-COP (loading control) antibodies. **A** Effects of varying amounts of mutant SR-BI and SR-BII expressing plasmids in the transfection together with either 0% (−) or 50% (+) PDZK1 expressing plasmid. **B** Effects of varying amounts of PDZK1 expressing plasmid transfected together with 1% SR-BI Δ509 expressing plasmid.

Figure 4A shows the effects of coexpression in COS cells of PDZK1 on the levels of expression of these three SR-BI variants as a function of the amounts of variant vector transfected (compare to that of wild-type SR-BI in Figure 2A). Unexpectedly, the results for all three variants - Δ509, ΔC-term and SR-BII – were similar to those for wild-type SR-BI. PDZK1 had essentially no effect on the amount variant receptor proteins when the ratio of SR-BI-to-PDZK1 was 50∶50 (two left lanes), yet there was a reproducible increase in the amount of variant receptors in the presence of PDZK1 when lower levels of variant receptor vector were used (right lanes in Figure 4A). [The ε-COP loading controls, which are shown in Figure S2, demonstrate that equivalent amounts of sample were loaded.] In addition, Figure 4B shows that the amount of Δ509 protein increased as the PDZK1 vector increased, similar to the results for wild-type SR-BI (Figure 2B).

We conclude that the PDZK1-dependent increase in SR-BI expression described here in COS cells, and possibly HEK293 cells, is not likely to occur via the same mechanism as that which occurs in hepatocytes *in vivo*, because the PDZK1-dependence in cultured cells did not depend on the C-terminus of SR-BI, as it does *in vivo*. [The mechanism of the apparently artifactual PDZK1-dependence of SR-BI in COS cells remains unknown.] Unfortunately, this COS cell expression system does not provide a robust system to study the mechanism by which PDZK1 controls hepatic SR-BI localization and abundance *in vivo* [36]. The relevance of our findings with COS cells to the earlier reports of PDZK1 dependent SR-BI expression in cultured cells [26], [35], [51] is unclear. Differences in cell types and culture conditions may significantly influence the mechanism by which PDZK1 affects SR-BI expression *in vitro*. Nevertheless, our results suggest that it may be prudent to validate any *in vitro* cell culture system used to study the mechanism of PDZK1-dependent SR-BI expression using controls such as the SR-BI variants Δ509, ΔC-term and SR-BII. Additional studies will be required to develop cultured hepatocyte or cultured cell line methods that will permit the elucidation of the mechanism by which PDZK1 controls SR-BI hepatic protein expression.

## Materials and Methods

### Materials

High density lipoprotein (HDL) was isolated at the Massachusetts Institute of Technology from human plasma as described previously [55] using a protocol for obtaining human plasma with donor written informed consent that was approved by the Massachusetts Institute of Technology’s Committee on the Use of Humans as Experimental Subjects (protocol #0403000011). The rabbit polyclonal anti-SR-BI antibody, which recognizes the carboxy- terminus of SR-BI (mSR-BI^495^), was developed in our laboratory [5], [7]. Another rabbit polyclonal anti-SR-BI antibody, which recognizes extracellular loop of SR-BI (KKB-1) was described previously and kindly provided by Dr. Karen F. Kozarsky [56]. The rabbit polyclonal anti-mouse PDZK1 antibody and rabbit polyclonal anti-ε-COP antibody were developed in our laboratories [42], [57]. A monoclonal anti-rat PDZK1 antibody was kindly provided by Dr. Hiroyuki Arai (Tokyo University) [35]. Mouse SR- BII (alternatively spliced form) cDNA was kindly provided by Dr. Deneys R. van der Westhuyzen (University of Kentucky Medical Center) [16]. The deletion mutants of SR-BI were generated by PCR. All cDNA fragments were cloned into a mammalian expression vector, pcDNA3.1 (Invitrogen, CA) and used for transfection into COS or HEK293 cells.

### Isolation and Culture of Primary Hepatocytes

Primary mouse hepatocytes were isolated from 8 week old Male C57BL/6 mice using a two-step collagenase perfusion protocol described in detail by Martinez *et. al.* [58]. Perfusions yielded initial cell viability (trypan blue exclusion) of about 80% and final cell viability after Percoll gradient centrifugation of about 90%. Hepatocytes harvested at time ‘0′ were pelleted and then lysed in lysis buffer A (10 mM Tris-HCl (pH7.4), 150 mM NaCl, 1% NP-40, 1 mM EDTA, and proteinase inhibitor cocktail (cOmplete, Roche, 20x stock solution prepared as 1 tablet/2 ml of water)) and the lysates were frozen at −80°C for subsequent immunoblotting analysis.

A description of the development of the conditions used in this study to culture primary mouse hepatocytes beyond time ‘0′ will be described in detail elsewhere (Buck et al, unpublished data). This method uses a ‘sandwich’ culture procedure in which cells are plated onto either collagen adsorbed directly onto the culture dish (Buck et al, unpublished data) or a collagen type I gel (this study). Four hours after plating, the cells are overlayed with a thin layer of Matrigel (BD Biosciences) with cell seeding densities and medium volume adjusted to provide a physiologically-relevant oxygen tension (i.e., an oxygen tension comparable to the central zone in the liver sinusoid) at the cell surface) (Buck et al, unpublished). These conditions using plating on adsorbed collagen result in the long-term retention of function as assessed by albumin secretion and sensitivity to killing by acetaminophen (Buck et al, unpublished). In this study the mouse hepatocytes were seeded onto collagen gels in the wells of 6-well plates at a density of 80,000 cells/cm^2^ in 1.3 ml William’s E medium (1.3 mm medium depth) supplemented with 10% FBS, 1 mg/ml aprotinin, 1X antibiotic/antimycotic (Gibco), 10 mM HEPES, 0.1 µM dexamethasome, 10 µg/ml ITS (Roche), 2 mM glutamax (Sigma-Aldrich) (Mouse Seeding Medium). The collagen gels (1.6 mg/mL of collagen I in PBS with 2 g/L glucose and 3.7 g/L sodium bicarbonate) were prepared as described previously [59]. Mouse Seeding Medium was replaced after 4 hours with 1.3 mL of serum-free Mouse Maintenance Medium (Mouse Seeding Medium without FBS +3% Matrigel). Medium was either collected (albumin secretion assay) or discarded and replaced with 1.3 mL fresh Mouse Maintence Medium (without Matrigel) every 24 hours thereafter. The 3% Matrigel supplemented in Mouse Maintenance Medium was added again on day 4. Cells were harvested for analysis after 1, 4, and 7 days of culture. In this study we have shown that plating on a collagen type I gel maintains albumin secretion at rates that were as good as or better than plating on adsorbed collagen (Buck et al, unpublished). The albumin secretion reached a plateau of approximately 45 µg/cm^2^/day and was maintained without decline over 7 days in culture (see Figure S3). We have also performed preliminary analysis of the sensitivity of mouse hepatocytes cultured for four days using the collagen gel to killing by acetaminophen (EC_50_ ∼20 mM) and found it to be similar to that when the cells were plated onto adsorbed collagen (Buck et al, unpublished).

### Ethics Statement

All experiments using animals described here were performed in strict accordance with NIH and Massachusetts Institute of Technology guidelines and approval of the Committee on Animal Care at the Massachusetts Institute of Technology (approved protocol numbers 0111-001, 0209-015 and 0212-015). Isolation of human plasma with donor written informed consent followed a protocol that was approved by the Massachusetts Institute of Technology’s Committee on the Use of Humans as Experimental Subjects (protocol #0403000011).

### Culture and Transfection of Cell Lines

COS M6 cells (Biology Department, Massachusetts Institute of Technology, Cambridge, MA) [56] and HEK293 (gift from H. Lodish, Biology Department, Massachusetts Institute of Technology and the Whitehead Institute, Cambridge, MA) cells were cultured in DMEM medium (Mediatech, Manassas, VA) supplemented with 10% (v/v) fetal bovine serum and antibiotics (100 IU/ml penicillin and 100 mg/ml streptomycin) (DMEM/FBS). The transfections were carried out using Lipofectamine 2000 reagent (Invitrogen) according to the manufacturer’s protocol. Briefly, on day 0, cells (500,000 cells/well for COS cells, and 2,000,000 cells/well for HEK293 cells) were plated in the wells of 6-well plates in DMEM/FBS. On day 1, cells were transfected with the plasmids (4 µg total DNA per well) using Lipofectamine 2000 in Opti-MEM I reduced serum medium (Invitrogen). The amounts of each DNA plasmid used for co-transfection experiments are indicated as the percentage of the total amount of DNA (4 µg) transfected. Empty vector (pcDNA 3.1, Invitrogen) was added when necessary to bring the total amount of DNA to 4 µg (100%). For immunoblot analyses, on day 2, the medium was replaced with fresh DMEM/FBS and then the cells were harvested for analysis on day 3. For cholesteryl ester uptake activity assays (see below), the transfected cells were harvested, counted and transferred to 24-well plates (100,000 cells/well in DMEM/FBS) on day 2 and assays were performed on day 3.

### Measurement of Albumin

Conditioned medium collected after 24 hours was stored at −20°C. Samples were thawed and the concentration of mouse albumin was determined using a mouse albumin enzyme-linked immunosorbent assay (ELISA, Bethyl Laboratories) following the manufacturer’s suggested protocol.

### Quantitative RT-PCR

Primary mouse hepatocytes were harvested on day 0 or cultured for the indicated times using the collagen gel sandwich method (see above). The cells were washed once with 1 mL PBS. Total RNA was isolated with the RNeasy kit following the manufacturer’s instructions (Qiagen, CA), and cDNA was prepared using reverse transcriptase III (Invitrogen) as described [60]. MGTP, a form of quantitative real-time PCR, was used to determine mRNA copy numbers per cell [61], [62]. The number of mRNA copies per cell was calculated by normalization to 18S rRNA abundance, assuming that, on average, cells express ∼10^6^ 18S-rRNA copies. Primer sets used for PCR amplification are: SR-BI, GTCATGATCCTCATGGTGCC and TTCGAAGAAGTAGACAGACAAGT; and PDZK1, GCTCAGGATCAATGGTGTCTTTG and CCATCCAGGACCAGCAGAGT. Hepatocytes were harvested from three mice each day on three different days and the mRNA values for duplicate wells of hepatocytes from each mouse were measured. The values shown in Figure 1B are averages of all of the data from mice from two different days. Similar results were obtained from mice harvested on the third day.

### Immunoblot Analysis

The cells were lysed in lysis buffer A and samples (20 µg protein) were fractionated using 10% SDS-PAGE and transfered onto polyvinylidene difluoride (PVDF) membranes. Detection was performed using primary polyclonal or monoclonal antibodies and HRP-conjugated secondary antibodies together with an enhanced chemiluminescence (ECL) plus kit (GE Healthcare) according to the manufacturer’s protocol. The results shown are representative of multiple, independent experiments.

### SR-BI-mediated Cholesteryl Ester Uptake Assay

HDL was labeled with [^3^H]cholesteryl oleate ([^3^H]CE) (Perkin-Elmer no. NET746L) as previously described [55]. COS cells were transfected and plated as described above and on day 3 the cells were washed twice with prewarmed (37°C) DMEM. We then added 500 ml/well of fresh assay media (DMEM with 100 IU/ml penicillin and 100 µg/ml streptomycin plus 0.5% (w/v) bovine serum albumin (BSA) containing 10 µg protein/mL of [^3^H]CE-HDL without or with a 40-fold excess of unlabeled HDL to determine non-specific uptake. Cells were incubated for 2 h in a 5% CO_2_ incubator at 37°C. The radioactive assay media were then removed and the cells washed rapidly two times with ice-cold wash buffer 1 (0.9% NaCl, 50 mM Tris-HCl, pH 7.4) containing 2 mg/mL BSA and once with wash buffer 1 without BSA. Lipids were extracted from the cells into 1 mL of 2-propanol for 30 min at room temperature, and all of which was then subjected to liquid scintillation counting. The remaining lipid-depleted cell extracts were lysed in 500 ml of 0.1 N NaOH, and protein content was determined by the method of Lowry [63]. The amounts of cell-associated [^3^H]CE (uptake) are expressed as the equivalent amount of[^3^H]CE-HDL protein (ng of the protein component of the lipoprotein/mg of cell protein). The values presented represent SR-BI specific uptake and were calculated as the differences between the average of 4 replicates of total cell associated uptake determined in the absence of unlabeled HDL minus the average of duplicate determinations in the presence of 40-fold excess unlabeled HDL (nonspecific uptake).

## Supporting Information

Figure S1 **Effects of PDZK1 co-transfection on SR-BI protein levels in HEK293 cells.** HEK293 cells were plated into the wells of 6 well plates (2,000,000 cells/well) on day 0 and transiently transfected with a total of 4 µg DNA (100%)/well on day 1 using the indicated plasmids encoding SR-BI, PDZK1 and an empty vector at the indicated relative concentrations (%). On day 3 the cells were harvested, lysed, and lysates (20 µg protein) were subjected to SDS-PAGE and immunoblotting with polyclonal anti-SR-BI (mSR-BI^495^) and polyclonal anti-ε-COP (loading control) antibodies. Immunoblots show the effects of varying amounts of SR-BI expressing plasmid in the transfection with either 0% (-) or 50% (+) PDZK1 expressing plasmid.
(TIF)

Figure S2 **Loading controls (ε-COP) for the experiment shown in** **Figure 4A** **(Effects of PDZK1 co-transfection on mutant SR-BI and SR-BII protein levels in COS cells.).** Polyclonal polyclonal anti-ε-COP antibody was used as the primary antibody for immunoblotting as a loading control for the experiment shown in Figure 4A.
(TIF)

Figure S3 **Time course of albumin secretion in cultured primary mouse hepatocytes.** Hepatocytes were isolated from the livers of wild-type mice and plated and maintained for 7 days in a collagen gel/Matrigel sandwich culture as described in Materials and Methods. Rate of albumin secretion was determined using an Elisa assay as described in Materials and Methods.
(TIF)
