# Supplementary figures and images for: Challenges in Using Cultured Primary Rodent Hepatocytes or Cell Lines to Study Hepatic HDL Receptor SR-BI Regulation by Its Cytoplasmic Adaptor PDZK1
Source: PLoS One. 2013 Jul 23;8(7):e69725. doi: 10.1371/journal.pone.0069725 (PMC3720616; doi:10.1371/journal.pone.0069725)

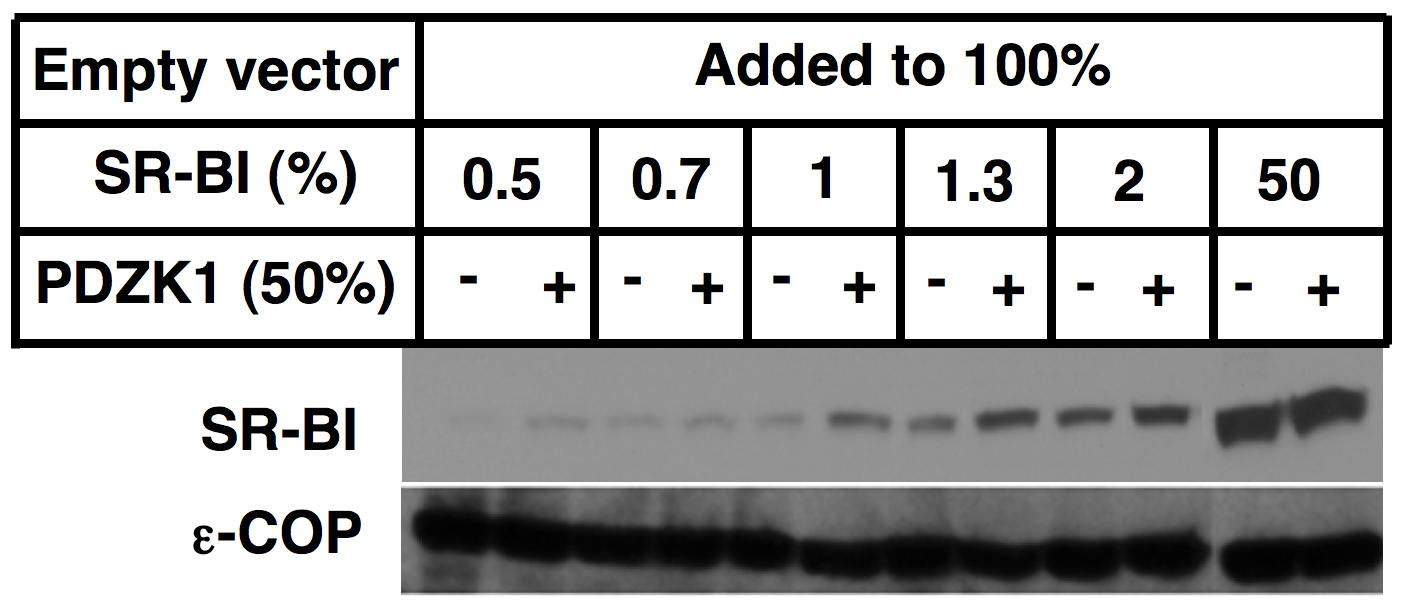

Supplement: Figure S1 — Effects of PDZK1 co-transfection on SR-BI protein levels in HEK293 cells. HEK293 cells were plated into the wells of 6 well plates (2,000,000 cells/well) on day 0 and transiently transfected with a total of 4 µg DNA (100%)/well on day 1 using the indicated plasmids encoding SR-BI, PDZK1 and an empty vector at the indicated relative concentrations (%). On day 3 the cells were harvested, lysed, and lysates (20 µg protein) were subjected to SDS-PAGE and immunoblotting with polyclonal anti-SR-BI (mSR-BI495) and polyclonal anti-ε-COP (loading control) antibodies. Immunoblots show the effects of varying amounts of SR-BI expressing plasmid in the transfection with either 0% (-) or 50% (+) PDZK1 expressing plasmid. (TIF) [file pone.0069725.s001.tif]

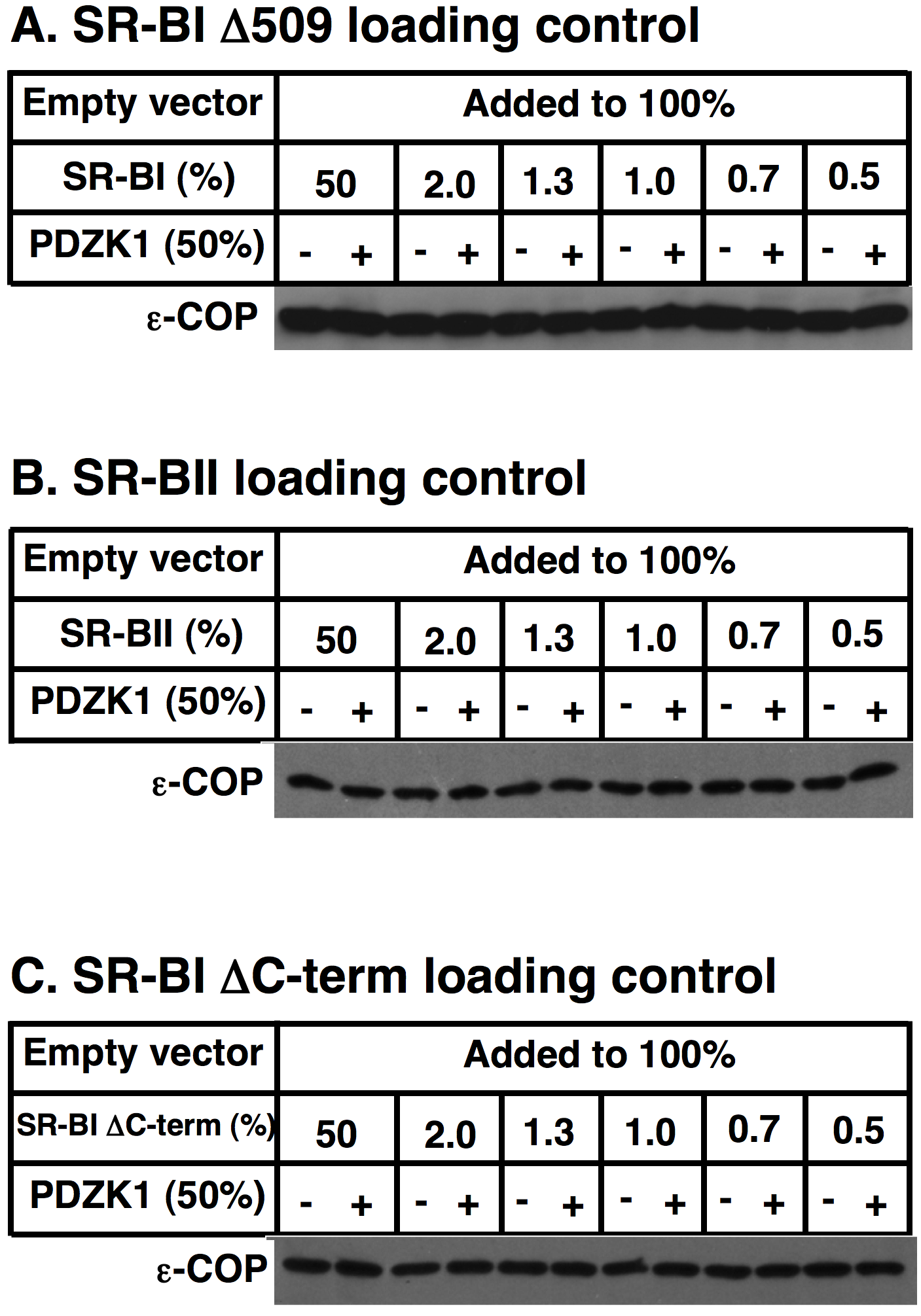

Supplement: Figure S2 — Loading controls (ε-COP) for the experiment shown in Figure 4A (Effects of PDZK1 co-transfection on mutant SR-BI and SR-BII protein levels in COS cells.). Polyclonal polyclonal anti-ε-COP antibody was used as the primary antibody for immunoblotting as a loading control for the experiment shown in Figure 4A. (TIF) [file pone.0069725.s002.tif]

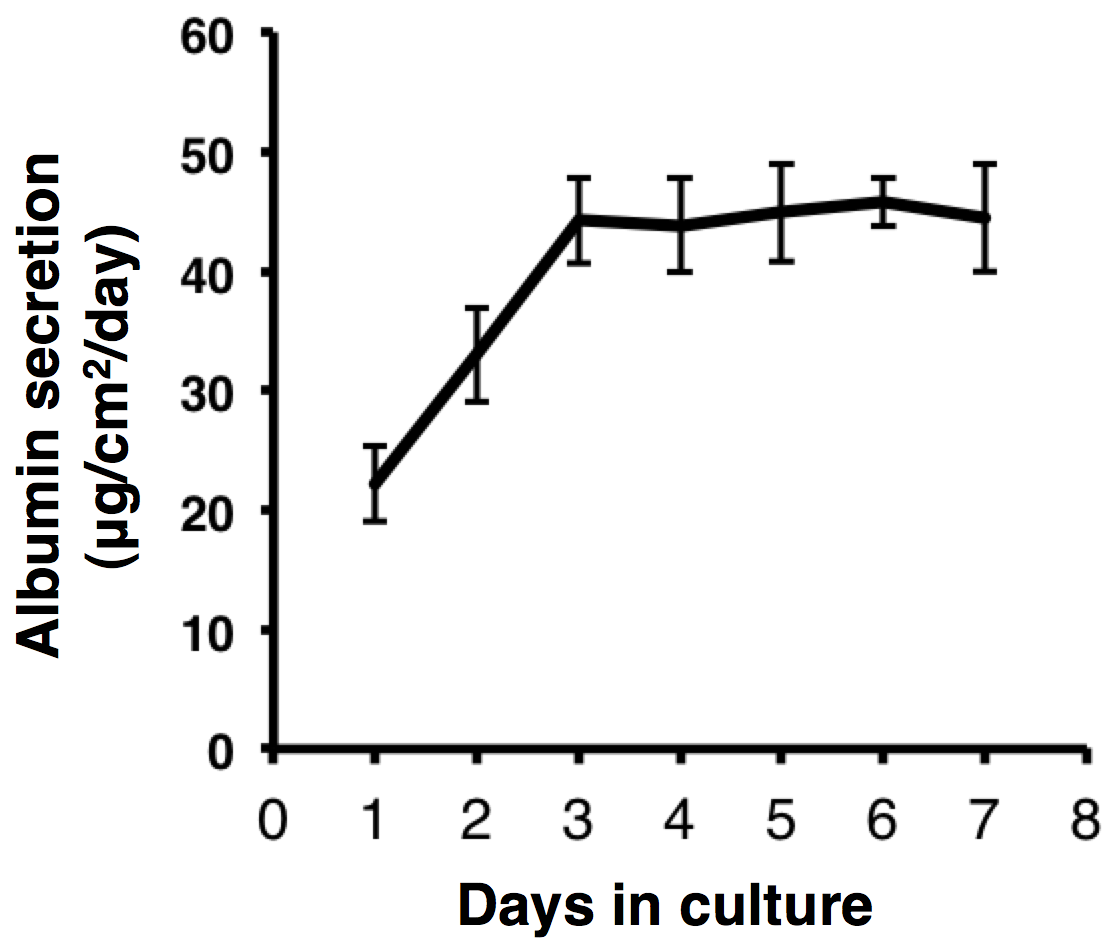

Supplement: Figure S3 — Time course of albumin secretion in cultured primary mouse hepatocytes. Hepatocytes were isolated from the livers of wild-type mice and plated and maintained for 7 days in a collagen gel/Matrigel sandwich culture as described in Materials and Methods. Rate of albumin secretion was determined using an Elisa assay as described in Materials and Methods. (TIF) [file pone.0069725.s003.tif]
